# Is there a "Scottish effect" for self reports of health? Individual level analysis of the 2001 UK census

**DOI:** 10.1186/1471-2458-6-191

**Published:** 2006-07-21

**Authors:** Frank Popham

**Affiliations:** 1 Research Unit in Health, Behaviour and Change, University of Edinburgh, Teviot Place, Edinburgh, EH8 9AG, UK

## Abstract

**Background:**

Scotland's overall health record is comparatively poor for a Western European country, particularly amongst people of working age. A number of previous studies have explored why this might be the case by comparing mortality in Scotland with England and Wales. A study in the 1980s showed that the higher prevalence of deprivation in Scotland accounted for Scotland's excess mortality risk. However, more recent studies suggest that deprivation now explains less of this excess. This has led to the suggestion that there is a yet unidentified "Scottish effect" contributing to Scotland's mortality excess. Recent research has also suggested that there could be an unidentified effect influencing Scotland's higher rate of heart disease. This paper explores whether there is also an unexplained Scottish excess, relative to England, in self reports of poor health.

**Methods:**

Data came from the individual Sample of Anonymised Records, a 3% random sample of the 2001 UK census. Using logistic regression models, self reports of health (limiting illness and general health) from the working age populations (aged 25 to 64) of Scotland and England were compared. Account was taken of people's country of birth. Stratified analysis by employment status allowed further exploration of Scotland's excess.

**Results:**

People born and living in Scotland reported higher levels of poor general health and limiting illness compared to people born and living in England. Adjustment for socioeconomic position and employment status largely explained the higher rates.

In the stratified analysis a Scottish excess was seen only amongst the economically inactive born and living in Scotland. For those in employment, people born and living in Scotland actually had slightly lower odds of reporting poor general health and limiting illness than people born and living in England.

**Conclusion:**

This analysis suggests that higher rates of poor self reported health in Scotland can be explained by differences in employment and socioeconomic position and so there is unlikely to be an unidentified "Scottish effect" for self reports of health. Scotland's excess of poor general health and limiting illness amongst the economically inactive is probably attributable to its economic and employment history.

## Background

Scotland's health is generally poor in comparison to other Western European countries, [1,2] with the worst all cause mortality rate amongst those of working age [2]. Why this is the case is the focus of on-going research. Studies have particularly focused on Scotland's health record in comparison to that of neighbouring UK countries.

Comparing mortality rates in the early 1980s in Scotland with England and Wales, Carstairs and Morris, using a census derived measure of local area deprivation, showed that the higher prevalence of deprivation in Scotland accounted for the Scottish mortality excess [3]. However, more recent ecological studies suggest that differences in deprivation, measured using the same scale (the Carstairs score), explained less of Scotland's excess mortality in the early 1990s and 2000s [1,4]. This unexplained excess in mortality has been called the "Scottish effect" [1,4].

Comparative research has also focused on Scotland's higher levels of morbidity in certain diseases. Mitchell *et al*. combined the 1998 Scottish and English national health surveys and showed that differences in individual risk factors (biological, behavioural, psychosocial and socioeconomic) between the populations of Scotland and England could only explain a small part of Scotland's higher prevalence of physician diagnosed heart disease [5]. This research suggests that there could be an unexplained effect influencing heart disease prevalence in Scotland [5].

A variety of explanations for the unexplained excess in both mortality and heart disease morbidity have been postulated. These include genetic differences, environmental differences, as yet unmeasured behavioural differences, psychosocial differences and a failure of existing measures to capture fully differences in socioeconomic and other risk factors [4-6].

In this paper, I assess whether there is also an unexplained Scottish excess in two self report measures of health using data from the 2001 census. Analysis of this census has shown that, overall, Scotland has a greater prevalence of poor general health amongst the working age population than England [7]. The focus of the present analysis is the degree to which this Scottish excess can be explained by differences in socioeconomic position (SEP) and employment.

Given that socioeconomic disadvantage across the lifecourse cumulatively impacts on people's mortality and morbidity risk, [8,9] and that migrants are likely to have different health and socioeconomic characteristics to non migrants, [10] account was taken not only of country of residence but also country of birth. Analysis of mortality data has shown that people born in England or Wales but living in Scotland have a lower standardised mortality rate (SMR) than people born and living in Scotland [11]. Moreover, people born in Scotland but living in England have higher SMR than people born and living in England [12]. Historically, Scotland has experienced high levels of outward migration [13] and until recently had net outward migration [14].

Account was taken of people's employment status as those not in employment have heightened mortality and morbidity risk independent of their SEP [8,15]. Scotland's economic history, and thus its employment history, is distinct from that of England [13]. Scotland's unemployment rate has historically been higher than England's, influencing the high levels of outward migration [13,16]. While the dramatic decline of traditional industries in the second half of the 20^th ^century changed employment patterns in both countries, the impact was, at a national level, particularly felt in Scotland where job loss was even more acute than in England [13].

The main research questions were thus:

• Taking account of country of birth, do people living in Scotland report higher rates of poor general health and limiting illness than people living in England?

• To what degree is Scotland's excess in poor general health and limiting illness explained by differences in employment and socioeconomic position?

• Is Scotland's excess in poor general health and limiting illness seen in all employment status groups?

## Methods

### The sample

The 2001 Samples of Anonymised Records (SARs) are a collection of random samples – created from individual and household level census data – made available to the research community to allow micro level analysis of the UK census. The samples are all anonymised and procedures are in place to ensure that individuals cannot be identified. Here the 2001 individual licensed SAR is used. This is a 3% sample of the 2001 UK census incorporating 1,843,525 individuals. Details of the individual SAR's methodology have been published elsewhere [17]. While the statistical office of each UK country is responsible for conducting their own census there is much co-ordination, making the individual SAR an attractive dataset to compare the health of individuals in Scotland and England.

### Variables

#### Demographics

As the focus was on those of working age, the sample was limited to 25 to 64 year olds not living in communal establishments and not identifying themselves as full time students. Age in the individual SAR was precoded, as follows, before the data were released (25 to 29, 30 to 44, 45 to 59, 60 to 64). The derived country of residence and country of birth variable has four categories (born and living in England, born in Scotland but living in England, born in England but living in Scotland, born and living in Scotland).

#### Self reported health

The 2001 UK census included two widely used self report health questions [18]. The presence of a limiting long term illness was assessed by the following question. "Do you have any long-term illness, health problem or disability which limits your daily activities or the work you can do?"

General health, captured for the first time in 2001 census, was assessed by the following question. "Over the last twelve months would you say your health has on the whole been: good, fairly good or not good?" For modelling purposes, responses were dichotomised not good health versus good/fairly good in line with other studies using this question [7,19].

#### Employment

People were categorised into three standard groups [20] based on their self reported employment status:

a) economically active and employed.

b) economically active (available for and seeking employment) but unemployed.

c) economically inactive (not seeking employment).

The economically inactive included those classifying themselves as being; retired, looking after the family or home, permanently sick or disabled, students not in full time education and people who did not identify with any of these categories.

#### Socioeconomic position

Although commonly used in UK health studies, occupational social class was not included as a measure of SEP in this study. This was because 12% of the sample was not given a social class independent of employment status. While all those in employment had a social class based on their present occupation, many of the economically inactive were not assigned a social class independent of their present employment status (being coded as long term unemployed, never worked, or not known for other reason). Alternatively, three measures of household level SEP were chosen because of their comparability across the nations. Housing tenure, housing conditions and household car ownership are commonly used measures of SEP that reflect, broadly, people's material circumstances [21] and that have been shown to be associated with health [21,22].

Following convention housing tenure was coded using the following categories; owned outright, owned with mortgage, privately rented and socially rented [21].

Following consultation with census users, a summary variable was derived by the census authorities from the questions covering housing conditions. Households living in deprived housing conditions were compared to all other households [23]. Deprived conditions were defined as living in either:

a) overcrowded accommodation (defined as households living in accommodation with less rooms than the household required given its size).

b) or a household sharing its dwelling (defined as a household whose accommodation was part of a converted or shared house and was not self contained behind its own door and was accessed by other households).

c) or a household sharing a bath or shower and toilet with another household.

d) or a household with no central heating.

The number of cars or vans owned or available for use in a household was categorised in the census as follows: none, one, two and three or more.

### Analysis

All analysis was conducted in Stata version 9 [24]. Initial descriptive analysis compared the distribution of the variables by country of residence and birth. Chi-square tests were used to assess associations.

Next, logistic regression models were used to further assess the association of country of residence and birth with limiting illness and general health. Models were fitted in two stages. The initial model was adjusted for age and sex only. At the next stage I additionally adjusted for SEP (using the three separate measures of SEP) and employment status.

To explore whether the Scottish excess in not good health and limiting illness was seen across all employment groups, models were then stratified by employment group. These models were initially age and sex adjusted and then additionally adjusted for the three measures of SEP. As men and women have very different rates and patterns of economic inactivity (14.5% of men, 30.4% of women) and employment (81.3% of men and 67.2% of women) the analysis for these employment groups was further stratified by sex to explore whether any Scottish excess in these employment groups was seen for both men and women.

## Results

From the SARs sample of 1,843,525 UK residents, the following exclusions were made.

a) The 143,607 (7.8%) residents of Wales and Northern Ireland.

b) Of those living in England and Scotland, the 189,604 (11.3%) people born outside of England and Scotland.

c) From the remainder, the 729,551 (48.3%) people who were not aged 25 to 64.

d) From the remainder, the 5080 people resident (0.7%) in communal establishments.

e) From the remainder, the 6798 (0.9%) full time students.

This left 768,885 people in the analysis sample. Unsurprisingly, the majority were born and living in England (87.3%), 2.1% were born in Scotland but living in England, 1.1% were born in England but living in Scotland and 9.5% were born and living in Scotland.

Table 1 gives the distribution of the variables of interest for each combination of country of residence and birth. Of the four groups, individuals born and living in Scotland were most likely to live in socially rented housing, to not have access to a car, to live in deprived housing conditions and to be unemployed or economically inactive.

Of those living in Scotland, people born in England were more likely to live in owner occupied housing, to not live in deprived housing conditions, to have car access and to be employed than people born in Scotland.

Of those living in England, people born in Scotland were slightly less likely to have car access and to live in an owned outright property than people born in England. However, there were no significant differences between the groups in terms of housing conditions or employment profile.

Limiting illness was most prevalent amongst people born and living in Scotland and least common amongst people born in England but living in Scotland. Those born and living in England had slightly lower prevalence of limiting illness than people born in Scotland but living in England. The same pattern was seen for the prevalence of not good general health. People born and living in Scotland were least likely to rate their general health as being good, with people born in England but living in Scotland having the highest rate. Of those living in England, those born in Scotland had a slightly higher rate of good general health than those born in England (table 1).

Taking account of age and sex differences, people born and living in Scotland were more likely to report a limiting illness and not good general health in comparison to people born and living in England (the reference category) (table 2). However, adjustment for SEP and employment status attenuated these associations dramatically (table 2). Those born in England but living in Scotland showed slightly lower odds of both not good general health and limiting illness than people born and living in England. Those born in Scotland but living in England showed similar odds, on both measures, to those born and living in England.

Stratifying the sample by employment status (table 3) showed that people living in Scotland who were in employment, whether born in Scotland or England, had slightly lower odds of reporting not good general health and limiting illness. Amongst the unemployed, a relatively small group, there was some evidence, particularly after adjustment for SEP, that unemployed people born and living in Scotland were less likely to report a limiting illness.

However, amongst the economically inactive, the pattern of results was similar to that seen for the whole sample, with people born and living in Scotland more likely to report not good general health and limiting illness, although after adjustment for SEP the association was attenuated somewhat. As those classifying themselves as permanently sick were very likely to report a limiting illness (96%) and not good general health (67%) the analysis of the economically inactive was rerun excluding the permanently sick. This made little difference to the pattern of results (table 3).

Table 4 shows that, for the economically inactive, there was an excess of not good general health and limiting illness for both women and men born and living in Scotland. Additionally, women born in Scotland but living in England had slightly higher odds of not good general health and limiting illness. For men born and living in Scotland, the excess in not good general health and limiting illness was explained by SEP adjustment; however there was a residual excess for economically inactive women who were born and living in Scotland.

For those in employment the pattern shown in table 3 of people born and living in Scotland having slightly lower odds of not good health and limiting illness, compared to people born and living in England, was seen for both sexes (results not shown).

**Figure 1:**
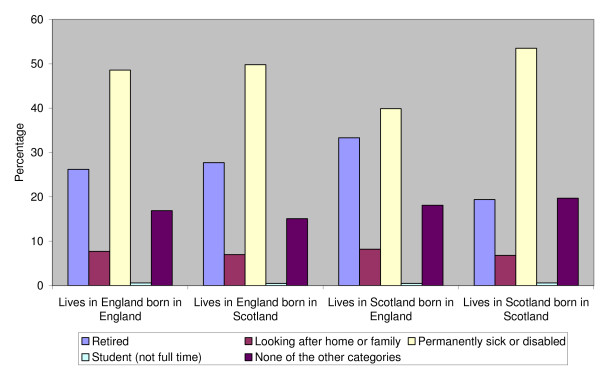
Classification of economically inactive men.

**Figure 2:**
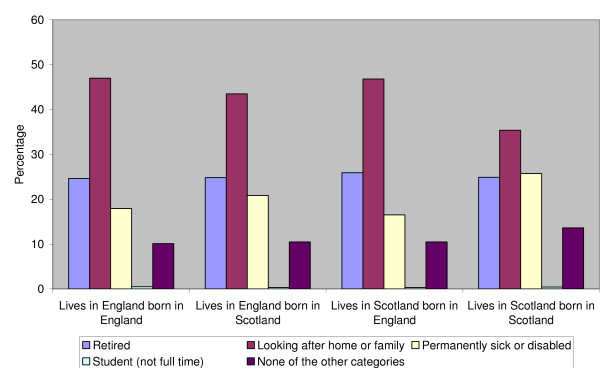
Classification of economically inactive women.

Given that any Scottish excess was only seen for economically inactive men and women who were born and living in Scotland, additional basic analysis was conducted to see whether classification of economic inactivity was also associated with country of birth and residence. There was a significant (p < 0.001) association between country of birth and residence and classification of economic inactivity for both men and women. Not only were economically inactive men and women born and living in Scotland more likely to be permanently sick, they were also more likely not to classify themselves in any of the categories, men born and living in Scotland were also less likely to be retired (Figures 1 and 2).

## Discussion

### Main findings

Working age people born and living in Scotland were, on average, more likely to report limiting illness and not good general health in comparison to people born and living in England. Adjustment for differences in SEP and employment status largely explained this association. This suggests that there is unlikely to be a universal and unidentified "Scottish effect" for self reported general health and limiting illness. Controlling for the impact of SEP or employment using either a single measure or measures from one time point in the lifecourse is unlikely to fully capture differences between populations [9,25]. In further analysis (available from the author) adjusting separately for only one of the measures of SEP, or just employment status, attenuated the relationship but not to the degree shown in table 2 when controlling for all these measures. It is likely that including further measures of SEP and employment status from across the lifecourse would have attenuated the relationship even further.

The stratified analysis supports the notion that SEP and employment status differences account for Scotland's excess in poor general health and limiting illness. Scotland's excess was only consistently seen amongst the economically inactive born and living in Scotland and this relationship was partly attenuated after controlling for the measures of SEP. People born and living in Scotland were most likely to be economically inactive and, amongst this employment group, most likely to classify themselves as permanently sick or not to identify with one of the census economic inactivity categories and (for men) least likely to be retired.

Why might people born and living in Scotland be more likely to be economically inactive? One possible reason is the lasting impact of industrial decline and labour market restructuring which has been particularly dramatic in Scotland since the 1980s [13]. In this period in Britain, not only did rates of employment for the manual social classes decline the most, the impact was felt greatest among those with a limiting illness [20]. So "a man has to be 'healthier' to remain employed in a manual rather than in a managerial, professional or clerical occupation" [20]. Moreover, there is strong evidence that unemployment figures, whether based on claimant counts or more widely accepted international definitions, seriously underestimate real levels of unemployment in old industrial areas because many (ill) long term unemployed with little prospect of finding work (and so regarding themselves as economically inactive) in these labour markets have been diverted from unemployment to sickness benefit [26]. Rates of sickness benefit have continued to rise for both men and women while unemployment has been on a overall downward trend in Britain [26]. In age and sex adjusted models (not shown) it was the economically inactive, rather than the unemployed, who had the highest odds of a limiting illness or not good general health in comparison to those in employment. This was true even after excluding people who described themselves as permanently sick or disabled.

Of course, the diversion from unemployment to sickness benefit has occurred in former industrial regions across Britain. However, a recent review of sickness benefit in Scotland shows that at the country level at least, Scotland has a higher percentage of the working age population claiming sickness benefit than England [27]. This review concludes that "Scotland's enormous number of incapacity claimants should really be interpreted as the legacy of twenty years of de-industrialisation and job destruction" [27].

In contrast to those who were economically inactive, people born and living in Scotland who were in employment seemed to be at slightly lower risk of poor health than people born and living in England. When accounting for SEP differences between the employed populations this association was actually strengthened slightly.

As discussed in the next section, there is the possibility that these results reflect national perceptions of poor health rather than actual differences in morbidity.

### Comparison with other studies

Ecological analysis of census results amongst those of working age showed that people living in Scotland overall reported worse general health than residents of England but compared to some regions of England and to the whole of Wales, Scotland's general health was actually better [7]. In the present study the analysis was extended by using individual level census data and additionally accounting for country of birth to compare the health of residents of Scotland and England. However, comparing self reports of health across countries is not unproblematic given possible variations in cultures and health expectations [28]. While self reported general health is a valid measure of health status, [29] and is predictive of future mortality risk independent of other risk factors, [30] it seems that the relationship between mortality and self reports of health varies in the countries of the UK, with people living in Scotland, in comparison to residents of England, self reporting better general health and less limiting illness than their mortality profile suggests they should [19,28].

Why this is the case is not clear. Given that Scotland's premature mortality rate is higher perhaps people living in Scotland have lower health expectations or perhaps cultural differences mean that they are more stoical. Recent work did not find any evidence that people in lower socioeconomic groups were more stoical when self reporting their health than people in higher groups, however the research was limited to Scotland [31]. Similar work comparing countries is required to help resolve the ongoing debate about the comparability of self reports of health across countries. In the meantime, the potential remains for self reports of health from Scotland to underplay the extent of any "Scottish effect" on morbidity.

Of course, there are major SEP differences in morbidity within countries and regions of the UK. In Scotland, social class differences in self reported health between the highest and lowest were the most acute in Britain for both men and women [7]. As Leon *et al*. argue there is a tendency, however, when discussing the possibility of a "Scottish effect" to conflate what is a between country issue with discussions of within country variations in inequalities in health [2].

The "Scottish effect" identified in relation to mortality remains unexplained. It would be fruitful to replicate the analysis here using nationally representative *individual *level data on mortality. The forthcoming (as of May 2006) census based Scottish Longitudinal Study, which links individual census data with mortality records, will allow direct comparison with the established ONS longitudinal study of English and Welsh censuses.

In ecological analysis of mortality data, an excess in Scotland existed in all deprivation categories from the most affluent to the most deprived, with differences in deprivation prevalence explaining less of the Scottish excess over time [1,4]. However, the possibility remains that established risk factors may explain the "Scottish effect". Scotland's mortality excess compared to England and Wales was greatest in the most deprived areas suggesting that if there is a "Scottish effect" influencing Scotland's mortality excess, it does not apply evenly across Scotland's population [2]. Moreover, the Carstairs score fails to explain all the current variation in mortality between English regions [2]. There is also evidence that another deprivation index largely explains regional mortality variation in England [32].

Future analysis should also explore cause specific mortality as well as all cause mortality as Scotland's comparative position in Western Europe varies by disease type [2]. For example, Mitchell *et al*. explored ischemic heart disease prevalence as Scotland's record is particularly poor for this disease [5].

### Strengths and limitations

To the best of the author's knowledge, this is the first study to compare rates of self reported general health and limiting illness in Scotland and England using individual, rather than ecological, census data. The individual SAR provided a large random sample of a whole population survey. Importantly, it allowed not just the exploration of between country of residence differences but also allowed account to be taken of country of birth.

Of course, using country of birth was a crude way to identify inter-country migration (for example, no information was available on when a person moved or why). The census does record the address of individuals who moved in the last year, however the level of inter country migration in one year was, relatively, small. As this study was cross-sectional it was not possible to explore issues of causality in this sample.

## Conclusion

Scotland's excess, compared to England, in rates of self reported not good general health and limiting illness amongst the working age population was largely explained by accounting for differences in SEP and employment between the two countries. In stratified analysis, an excess of poor general health and limiting illness was only seen amongst the economically inactive born and living in Scotland. People living in Scotland in employment were actually slightly less likely to report not good general health and limiting illness. These results are particularly interesting as the "Scottish effect" is part of Scotland's policy discourse on health. For example, the Chief Medical Officer's 2005 report on Scotland's health devotes its initial chapter to the "Scottish effect" and its possible causes [6]. The Scottish excess in mortality remains unexplained. Further work using individual level mortality data is needed to investigate whether there is really a distinct "Scottish effect" on mortality.

## Competing interests

The author(s) declares that he has no competing interests.

## Authors' contributions

FP is the sole author.

## Pre-publication history

The pre-publication history for this paper can be accessed here:



## Figures and Tables

**Table 1:** Distribution of variables by country of residence and birth (column percentages)

	*Lives in England born in England*	*Lives in England born in Scotland*	*Lives in Scotland born in England*	*Lives in Scotland born in Scotland*	*All*
**Total Number**	671187	16324	8046	73328	768,885
					
	*%*	*%*	*%*	*%*	*%*
Age***	^###^		^++^		
25–29	12.0	9.1	10.9	10.9	11.8
30–44	42.6	39.8	43.7	42.2	42.6
45–59	36.1	41.5	36.5	36.8	36.3
60–64	9.3	9.6	8.9	10.1	9.4
Sex***	^###^		^++^		
Male	49.5	51.4	49.9	48.1	49.4
Female	50.5	48.6	50.1	51.9	50.6
Tenure***	^###^		^+++^		
Owned outright	22.1	18.4	21.3	17.7	21.6
Owned with mortgage	54.9	53.6	53.5	52.9	54.7
Privately rented	8.7	13.7	13.1	6.5	8.6
Socially rented	14.3	14.3	12.1	22.9	15.1
Number of cars***	^###^		^+++^		
No car	13.2	16.4	12.4	21.0	14.0
1 car	41.7	40.4	44.6	45.1	42.0
2 cars	35.0	34.3	35.2	27.6	34.3
3 cars or more	10.2	8.9	7.8	6.3	9.7
Housing conditions***			^+++^		
Not deprived	88.2	87.8	89.8	84.1	87.8
Deprived	11.8	12.2	10.2	15.9	12.2
Economic activity***			^+++^		
Employed	74.6	75.0	74.8	69.9	74.2
Unemployed	3.2	3.1	3.7	3.9	3.3
Economically inactive	22.2	21.9	21.5	26.2	22.5
Limiting long term illness***	^##^		^+++^		
No	84.5	83.7	86.1	80.8	84.1
Yes	15.5	16.3	13.9	19.2	15.9
General health over last twelve months***	^###^		^+++^		
Good	67.4	68.2	72.2	65.3	67.3
Fairly good	23.7	22.0	19.6	23.2	23.6
Not good	8.9	9.8	8.2	11.5	9.1

Chi-square test (comparing all groups), * p < 0.05, ** p < 0.01, *** p < 0.001.

Chi-square test (comparing people living in England only), ^# ^p < 0.05, ^## ^p < 0.01, ^### ^p < 0.001.

Chi-square test (comparing people living in Scotland only), ^+ ^p < 0.05, ^++ ^p < 0.01, ^+++ ^p < 0.001.

**Table 2:** Self reported health by country of birth and residence (odds ratios and 95% CIs)

	**Limiting illness**	**Not good health**
Age & sex adjusted		
*Lives in England born in England*	1	1
*Lives in England born in Scotland*	1.00 (0.96 to 1.05)	1.06* (1.00 to 1.11)
*Lives in Scotland born in England*	0.88*** (0.82 to 0.93)	0.91* (0.84 to 0.98)
*Lives in Scotland born in Scotland*	1.28*** (1.26 to 1.31)	1.31*** (1.28 to 1.34)
+SEP and employment status adjusted^1^		
*Lives in England born in England*	1	1
*Lives in England born in Scotland*	0.97 (0.93 to 1.02)	1.02 (0.96 to 1.08)
*Lives in Scotland born in England*	0.87*** (0.81 to 0.94)	0.92 (0.85 to 1.01)
*Lives in Scotland born in Scotland*	1.05*** (1.02 to 1.07)	1.04** (1.01 to 1.07)

^1 ^Adjusted for age, sex, tenure, housing condition, car ownership and employment status.

* p < 0.05, ** p < 0.01, *** p < 0.001.

**Table 3:** Self reported health by country of birth and residence stratified by employed status (odds ratios and 95% CIs)

	**Limiting illness**	**Not good health**
**Employed **(age & sex adjusted)		
*Lives in England born in England (n = 501007)*	1	1
*Lives in England born in Scotland (n = 12238)*	0.97 (0.90 to 1.04)	1.02 (0.93 to 1.12)
*Lives in Scotland born in England (n = 6020)*	0.85** (0.76 to 0.94)	0.91 (0.78 to 1.05)
*Lives in Scotland born in Scotland (n = 51223)*	0.91*** (0.88 to 0.94)	0.92*** (0.87 to 0.96)
+SEP adjusted^1^		
*Lives in England born in England*	1	1
*Lives in England born in Scotland*	0.95 (0.88 to 1.02)	0.99 (0.90 to 1.09)
*Lives in Scotland born in England*	0.85** (0.77 to 0.95)	0.91 (0.78 to 1.05)
*Lives in Scotland born in Scotland*	0.86*** (0.83 to 0.90)	0.85*** (0.81 to 0.89)
		
**Unemployed **(age & sex adjusted)		
*Lives in England born in England (n = 21377)*	1	1
*Lives in England born in Scotland (n = 506)*	0.90 (0.71 to 1.15)	0.94 (0.69 to 1.29)
*Lives in Scotland born in England (n = 295)*	0.99 (0.73 to 1.35)	0.88 (0.57 to 1.35)
*Lives in Scotland born in Scotland (n = 2886)*	0.89* (0.80 to 0.99)	1.02 (0.89 to 1.17)
+SEP adjusted^1^		
*Lives in England born in England*	1	1
*Lives in England born in Scotland*	0.88 (0.70 to 1.12)	0.90 (0.66 to 1.23)
*Lives in Scotland born in England*	1.01 (0.74 to 1.38)	0.90 (0.58 to 1.38)
*Lives in Scotland born in Scotland*	0.84** (0.75 to 0.94)	0.96 (0.84 to 1.10)
		
**Economically inactive **(age & sex adjusted)		
*Lives in England born in England (n = 148803)*	1	1
*Lives in England born in Scotland (n = 3580)*	1.06 (0.99 to 1.14)	1.11** (1.03 to 1.19)
*Lives in Scotland born in England (n = 1731)*	0.86** (0.77 to 0.95)	0.91 (0.81 to 1.02)
*Lives in Scotland born in Scotland (n = 19219)*	1.47*** (1.42 to 1.52)	1.34*** (1.30 to 1.38)
+SEP adjusted^1^		
*Lives in England born in England*	1	1
*Lives in England born in Scotland*	1.01 (0.94 to 1.09)	1.05 (0.97 to 1.13)
*Lives in Scotland born in England*	0.88* (0.79 to 0.98)	0.94 (0.84 to 1.05)
*Lives in Scotland born in Scotland*	1.23*** (1.19 to 1.27)	1.13*** (1.09 to 1.17)
		
**Economically inactive excluding permanently sick **(age & sex adjusted)		
*Lives in England born in England (n = 107889)*	1	1
*Lives in England born in Scotland (n = 2465)*	1.02 (0.93 to 1.12)	1.06 (0.95 to 1.20)
*Lives in Scotland born in England (n = 1317)*	0.95 (0.83 to 1.08)	0.97 (0.82 to 1.15)
*Lives in Scotland born in Scotland (n = 12422)*	1.44*** (1.38 to 1.50)	1.38*** (1.31 to 1.46)
+SEP adjusted^1^		
*Lives in England born in England*	1	1
*Lives in England born in Scotland*	1.00 (0.91 to 1.10)	1.04 (0.92 to 1.17)
*Lives in Scotland born in England*	0.98 (0.86 to 1.12)	1.00 (0.85 to 1.19)
*Lives in Scotland born in Scotland*	1.23*** (1.18 to 1.29)	1.14*** (1.08 to 1.21)

^1 ^Adjusted for age, sex, tenure, housing condition and car ownership.

* p < 0.05, ** p < 0.01, *** p < 0.001.

**Table 4:** Self reported morbidity by country of birth and residence for the economically inactive stratified by sex (odds ratios and 95% CIs)

	**Limiting illness**	**Not good health**
**Economically inactive – Men **(age adjusted)		
*Lives in England born in England (n = 46647)*	1	1
*Lives in England born in Scotland (n = 1276)*	1.02 (0.91 to 1.15)	1.06 (0.95 to 1.19)
*Lives in Scotland born in England (n = 552)*	0.73*** (0.61 to 0.86)	0.81* (0.68 to 0.97)
*Lives in Scotland born in Scotland (n = 6650)*	1.29*** (1.22 to 1.36)	1.15*** (1.09 to 1.21)
+SEP adjusted^1^		
*Lives in England born in England*	1	1
*Lives in England born in Scotland*	0.92 (0.82 to 1.04)	0.98 (0.87 to 1.10)
*Lives in Scotland born in England*	0.73*** (0.61 to 0.87)	0.82* (0.69 to 0.99)
*Lives in Scotland born in Scotland*	1.06 (1.00 to 1.12)	0.98 (0.93 to 1.04)
		
**Economically inactive – Women **(age adjusted)		
*Lives in England born in England (n = 102156)*	1	1
*Lives in England born in Scotland (n = 2304)*	1.09* (1.00 to 1.19)	1.14** (1.03 to 1.26)
*Lives in Scotland born in England (n = 1179)*	0.94 (0.83 to 1.06)	0.98 (0.85 to 1.14)
*Lives in Scotland born in Scotland (n = 12569)*	1.55*** (1.49 to 1.61)	1.47*** (1.41 to 1.53)
+SEP adjusted^1^		
*Lives in England born in England*	1	1
*Lives in England born in Scotland*	1.07 (0.98 to 1.17)	1.11* (1.01 to 1.23)
*Lives in Scotland born in England*	0.98 (0.86 to 1.11)	1.03 (0.89 to 1.19)
*Lives in Scotland born in Scotland*	1.30*** (1.25 to 1.36)	1.24*** (1.18 to 1.29)

^1 ^Adjusted for age, tenure, housing condition and car ownership.

* p < 0.05, ** p < 0.01, *** p < 0.001.
